# Seismic evidence of liquid water at the base of Mars’ upper crust

**DOI:** 10.1093/nsr/nwaf166

**Published:** 2025-04-25

**Authors:** Weijia Sun, Hrvoje Tkalčić, Marco G Malusà, Yongxin Pan

**Affiliations:** Key Laboratory of Planetary Science and Frontier Technology, Institute of Geology and Geophysics, Chinese Academy of Sciences, Beijing 100029, China; Research School of Earth Sciences, The Australian National University, Canberra, ACT 2601, Australia; Department of Earth and Environmental Sciences, University of Milano-Bicocca, Milan 20126, Italy; Key Laboratory of Planetary Science and Frontier Technology, Institute of Geology and Geophysics, Chinese Academy of Sciences, Beijing 100029, China

**Keywords:** Mars, liquid water, upper crust, low-velocity zone, receiver function inversion

## Abstract

Liquid water was abundant on Mars during the Noachian and Hesperian periods but vanished as the planet transitioned into the cold, dry environment we see today. It is hypothesized that much of this water was either lost to space or stored in the crust. However, the extent of the water reservoir within the crust remains poorly constrained due to a lack of observational evidence. Here, we invert the shear wave velocity structure of the upper crust, identifying a significant low-velocity layer at the base, between depths of 5.4 and 8 km. This zone is interpreted as a high-porosity, water-saturated layer, and is estimated to hold a liquid water volume of 520–780 m of global equivalent layer (GEL). This estimate aligns well with the remaining liquid water volume of 710–920 m GEL, after accounting for water loss to space, crustal hydration and modern water inventory.

## Teaser

Seismic inversion reveals a significant low-velocity anomaly at a depth of 5.4–8 km of Mars, providing the first seismic evidence of liquid water at the base of Mars' upper crust.

## INTRODUCTION

Liquid water plays a crucial role in regulating the habitability of Mars. Multiple lines of evidence from geomorphological and geological/geochemical observations have suggested that abundant liquid water once existed in the form of rivers, lakes and oceans during the Noachian and Hesperian [1]. However, as Mars transitioned to a colder and drier climate during the Amazonian period [2], the presence of liquid water significantly diminished. One of the greatest mysteries on Mars is where liquid water went.

A potentially significant source of water loss on Mars could have been the escape of liquid water into space, driven by the interaction of solar wind with the Martian atmosphere [3]. This process could have contributed substantially to the depletion of water on the planet. Atmospheric observations of the deuterium-to-hydrogen isotope ratio support the water escape mechanism [1,4]. Another primary mechanism for potential water loss is crustal hydration through irreversible chemical weathering, where water and/or hydroxyl are incorporated into minerals, i.e. water–rock interactions [4,5]. This mechanism is supported by both orbital and *in situ* observations, which indicate widespread distributions of chemical weathering [5].

An alternative mechanism suggests that liquid water was sequestered into the crust, potentially in deep aquifers [3,6]. While rock physics computations suggest that liquid water might be preserved in the middle crust [6], the extent of the crustal reservoir remains poorly constrained [7]. This uncertainty is primarily due to the lack of fine-scale structural observations extending to the base of the upper crust, or even into the middle crust.

Ground-penetrating radar (GPR), whether equipped on an exploration rover or an orbiter [8–12], is an efficient tool for investigating shallow structures. With the use of high frequencies in the MHz and GHz range, subsurface resolution can achieve a scale of 1 to 100 meters. However, due to the significant attenuation of electromagnetic waves with increasing depth, the maximum detection depth is limited to ∼100 meters for rover radar and a few kilometers (<5 km) for orbiter radar sounding, which does not provide complete illumination of the entire upper crust.

Alternatively, measuring the propagation velocity of seismic waves is an important tool for revealing the presence of low-velocity anomalies that may indicate the existence of liquid water in the upper crust. Several simplified crustal models were derived from the low-frequency receiver function data (0.1–1 Hz) from marsquakes [13–15] recorded by the InSight mission (Interior Exploration using Seismic Investigations, Geodesy and Heat Transport). Due to the vertical resolution limitation, these simplified models suggest that the crust comprises three layers, assuming that the velocity remains constant within each crustal layer.

**Figure 1.**
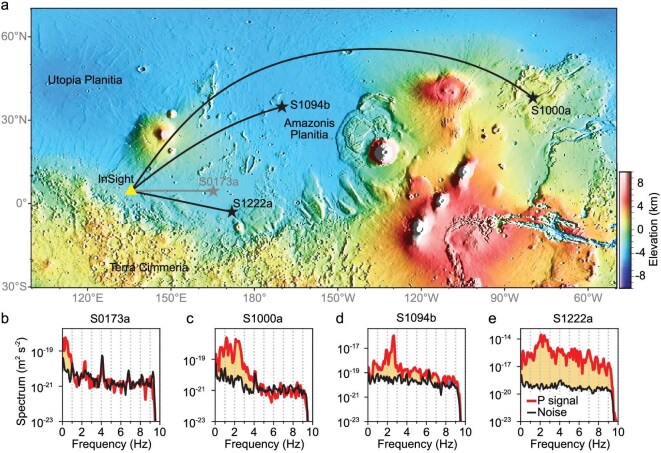
Locations and spectral analysis of marsquakes and impacts. (a) Location of the InSight station (yellow triangle) and events (black stars) used in this study. The S1000a and S1094b are two impact events, and the S0173a and S1222a are marsquakes that occurred at the crustal base or uppermost mantle. The black lines show the great circle of each event. (b)–(e) Spectral analysis of the four events for S0173a, S1000a, S1094b and S1222a. The yellow-shaded areas highlight P-wave signals (red lines) with strengths greater than the noise level (black lines). In contrast to the S0173a marsquake, which exhibits low frequencies (<1 Hz), the two impact events (S1000a, S1094b) and the largest tectonic marsquake (S1222a) contain rich higher-frequency (≥4 Hz) content. More detailed analyses are given in Data S1.

In this study, we analyze high-frequency seismic data (0.25–4 Hz; Fig. 1 and Text S1) from two meteorite impacts, S1000a and S1094b [16], and a marsquake event, S1222a [17], to significantly enhance the vertical resolution. For robust velocity inversion, we utilize two distinct inversion schemes, i.e. deterministic inversion and statistical Bayesian inversion with dimension fixed (see Methods), both aimed at investigating and validating the fine-scale structures of the upper crust. These are further verified via synthetic data. Our inversion results from both schemes reveal a significant shear-wave velocity anomaly at a depth of 5.4–8 km, raising a possibility of liquid water at the base of the upper crust.

## RESULTS AND INTERPRETATIONS

**Figure 2.**
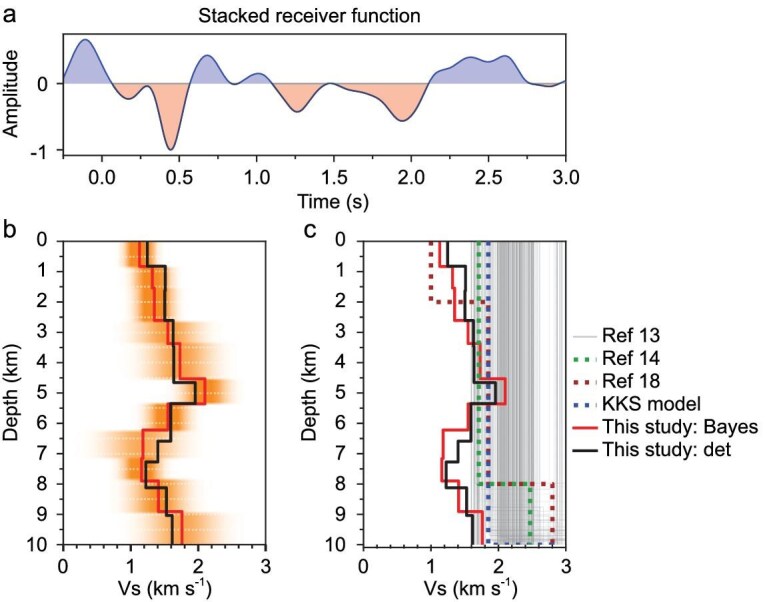
Inversion of velocity models. (a) The stacked receiver function for inversion. (b) The probability density function of the inverted shear wave velocity model from this study's true amplitude receiver function. The red line denotes the mean model of the Bayesian inversion, and the orange colors represent the posterior probability density function (PDF) of the Bayesian models. The black line indicates the model inverted from the deterministic scheme. (c) Models from previous studies using conventional receiver function and travel time inversion [13,14,18] for comparison to our models from the Bayesian and deterministic schemes. The crustal part of the KKS model is from ref. [15].

We calculate the high-frequency-preserved receiver functions for three events (S1000a, S1094b and S1222a) using the true amplitude receiver function method (see Methods for details). The stacked receiver function for inversion is shown in Fig. 2a. The details of data processing are provided in Data S2 and S3. To ensure the robustness of the inverted models, we implement two distinct inversion schemes: a deterministic scheme and a fixed-dimensional Bayesian scheme. These two methods are employed to obtain the high-resolution S-wave velocity structure of the upper crust in Fig. 2b. We present the velocity model from previous studies in Fig. 2c for comparison. As shown in the figure, the use of higher-frequency content significantly improves resolution, revealing three primary layers in the upper crust, in contrast to the single-layer structure identified in earlier models (Fig. 2c). The robustness of the inversion schemes from the stacked receiver function is further examined via a synthetic data set and by fitting individual receiver function in Data S4.

The model obtained from the deterministic scheme closely aligns with the mean mode of randomly selected models shown in the probability density function from the statistical Bayesian scheme in Fig. 2b. We identify a shallow discontinuity at a depth of 0.8 km, which corresponds to the previously estimated depth of 2 km [18]. This disparity results from our inverted velocity being higher than the estimation of ref. [18], as illustrated in Fig. 2b. The observed low velocity in this shallow region supports interpretations of a highly fractured layer [18]. A single ejecta zone [19] or ejecta and sediments interbedded with altered basalts [20] also explain the observed velocity.

The second layer exhibits a gradually increasing velocity structure from our inverted models. Both models from two inversion schemes indicate a consistent discontinuity at a depth of ∼5.4 km. This aligns with the results of 6.0 ± 2.4 km obtained from the joint inversion of low-frequency receiver functions and apparent shear wave velocity [21]. This layer exhibits a higher S-wave velocity, suggesting the presence of lithified sedimentary rocks or altered igneous rocks with some degree of porosity [22].

A prominent low-velocity layer is identified, with its lower boundary at a depth of 8 km, which aligns well with previously reported values of 8–10 km [13–15]. This discontinuity was interpreted as the base of the highly altered and fractured upper crust [23]. The S-wave velocity in this layer is as low as 1.2–1.5 km/s, approximately equal to that of the surface layer. However, based on the position of this layer, it is not reasonable to interpret this low velocity as indicative of the presence of sediments. Given the high porosity of altered basalts or igneous rocks, we examine the possibility that this low-velocity layer contains pure liquid water.

## DISCUSSION

### Enhancing resolution with adaptive parameters

**Figure 3.**
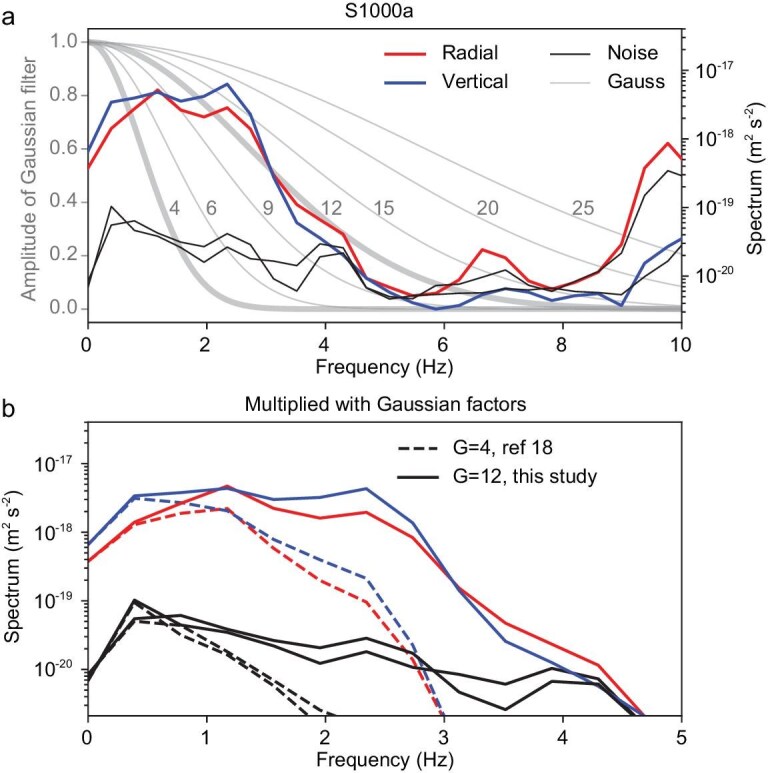
Illustration of the significance of selecting an appropriate low-pass Gaussian filter. (a) The amplitude of the Gaussian filter with different factors (gray lines) overlaid on the spectrum of S1000a (red and blue lines) and noise (black lines). (b) The spectrum filtered using a lower low-pass Gaussian filter factor of 4 (dashed lines) [18] and a higher low-pass factor of 12 (solid lines) (this study) for both signal (blue and red lines) and noise (black lines).

The crustal structures were mostly derived from the low-frequency (<1 Hz) receiver functions [13–15]. Due to the limited vertical resolution, the internal velocity variations within the upper crust are not well delineated in Fig. 2b. Using higher frequencies of 1–2 Hz, a shallow interface at a depth of 2 km has been identified [18]. However, these studies utilized a Gaussian factor of 4 to calculate the receiver function, which resulted in filtering out frequencies above 2 Hz carried by the events [18]. In Fig. 1c–e, the spectra of the selected events clearly show frequencies reaching 4 Hz or higher. Using a Gaussian factor of 4 primarily emphasizes the low-frequency content and attenuates the high-frequency content (dashed lines in Fig. 3b), thereby limiting the resolution. We adaptively select a Gaussian factor of 12 based on the spectral shape in Fig. 3a, which maximally preserves the high-frequency content (solid lines in Fig. 3b) and thus substantially enhances the resolution.

### Water ice or liquid water?

It has been proposed that one-third of Mars contains subsurface water ice, starting from just 1–2 meters below the surface and extending to a thickness of over 100 meters [24]. These substantial ice reserves are present in both hemispheres, spanning from mid-latitudes to the poles. Interestingly, water frost and ice have also been identified in low-latitude regions using data from orbital imaging spectrometers [25], however, the 15°S–15°N range appears to lack such deposits, likely due to thermal instability caused by higher surface temperatures [26].

**Figure 4.**
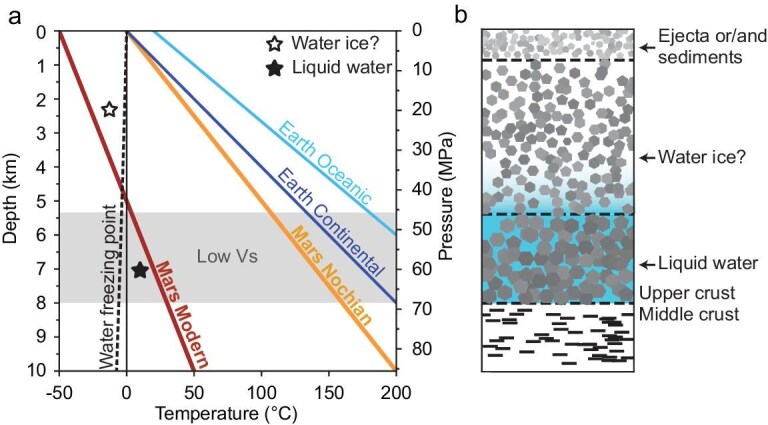
Thermal profiles and water storage model. (a) Temperature profiles for Earth and Mars adapted from ref. [28]. The water freezing point, varied with pressure, is shown by the black line (dashed line = pure water; continuous line = seawater). Specifically, the dark red line represents the modern temperature profile of Mars. The shaded layer denotes the low-Vs upper crustal base containing liquid water. (b) Schematic model for water storage in Mars’ upper crust.

The surface heat flow on Mars is relatively low, with an average value of 14–25 mW/m^2^ [27]. As a result, the temperature of the upper crust might often remain below the freezing point of liquid water. We therefore examine the temperature profile in the upper crust [28] in Fig. 4a. Observations from the temperature profile indicate that at depths shallower than 5 km, the temperature remains below the freezing point of water, implying that any water present at these depths likely exists as ice.

While the InSight lander, positioned near the equator, cannot have ice within 5 meters of the surface [29], the temperature profile (Fig. 4a) suggests that, if any, water ice could be present at depths shallower than 5 km. In contrast, at depths greater than 5 km, temperatures rise above the freezing point of water, permitting the presence of liquid water. Furthermore, the temperature and pressure conditions at the base of the upper crust (45–70 MPa, 10–30°C) do not reach supercritical levels, i.e. > 374°C and > 22.1 MPa for pure water, and > 406°C and > 29.8 MPa for seawater [30, 31]. As a result, these conditions permit the existence of pure liquid water. This reinforces the interpretation that the low-velocity anomaly is a sign of liquid water reservoirs.

### Liquid water reservoir: upper crust vs. middle crust

Computations from rock physics models hypothesized that the liquid water was sequestered in the middle crust at depths ranging from 11.5 km to 20 km, assuming an average porosity of 17% in the middle crust [6,19]. It is well known that the liquid water in the crust is stored in fractures or pores. However, it is unlikely that this porosity would extend downward to the deep crust in the view of gravity [32]. As depth increases, lithostatic loading and rock compaction can effectively close the pore spaces. Using reasonable estimates of the heat flux and other parameters such as thermal conductivity, the fracture/pore closure model suggests that porosity at depths deeper than 11 km has been removed [33]. Further, their model interpreted the velocity discontinuity at the depth range of 8–11 km [23] as the base of the porous layer (i.e. upper crust), which is consistent with our inverted velocity model shown in Fig. 2. Based on the fracture/pore closure model, it is likely that the liquid water is stored in the high-level fracture/pore layer at the base of the upper crust (see Fig. 4b), which corresponds to the significant low-velocity anomaly revealed by our inversion results.

### Liquid water volumes at the upper crust base

The total volume of surface liquid water early in Martian history was estimated to be equivalent to a global equivalent layer (GEL) of approximately 100 to 1500 meters [4,34]. The loss of liquid water occurred through three primary processes: escape into space, crustal hydration, and sequestration within the upper crust. Interestingly, the formation of valley networks on the Martian surface required at least 640 m GEL of water [35], which was ultimately lost or escaped through the processes mentioned above.

As listed in Table 1, the estimated volumes of liquid water lost to space and through crustal hydration were 10–200 m GEL and 550 m GEL, respectively [4,36]. The present-day water inventory in the atmosphere and as ice in the polar regions, or as subsurface ice, totals 20–40 m GEL [10,34,37]. However, there remains a significant volume of water, ∼710–920 m GEL, that cannot be accounted for. We interpret that this volume of water was sequestered into the base of the upper crust, a potential mechanism supported by our seismic observations of a prominent low-velocity anomaly at that location in Figs 2 and 4b.

**Table 1.** Integrated water volumes across different reservoirs and loss mechanisms. The remaining volume is calculated by subtracting the lower and upper limits of water volume for each component from the upper limit of the total water volume.

Items	Volume (m GEL)	References
Estimated total water volume	100–1500	[4, 34]
Modern water		
(in the atmosphere and as ice)	20–40	[10, 34, 37]
Water escaped into space	10–200	[4, 36]
Crustal hydration	550	[4]
Remaining volume	∼710–920	
Storage in the upper crust	520–780	This study

Existing seismic studies have revealed that the upper crust exhibits a high porosity ranging between 20% and 30% derived from velocity [14,22]. Gravity studies conducted beneath the InSight landing area suggest a lower porosity of around 12%–15% based on the density estimation [32]. Given that the estimation resolution of seismic velocity is generally considered higher than that of gravity density, we use the 20%–30% porosity range. This value is also confirmed by the scattering theory [22,38]. Given that the upper crust base is fully liquid-water-saturated and the InSight location is globally representative [6], the liquid volume preserved at the upper crust base is $thickness \times \textit{porosity} = 2.6km \times \,\,20{\mathrm{\% }}\,\,( {30{\mathrm{\% }}} ) = 520\,\,( {780} )$ m GEL. The value calculated from our observations aligns well with the missing liquid water volume of 710–920 m GEL, as estimated by other multidisciplinary studies (see Table 1).

It is important to note that the estimated water volume is anchored by the velocity structures beneath the InSight landing area, without accounting for potential lateral structural variations. This finding can be validated by future missions equipped with seismometers on Mars. Alternatively, extending this estimation to the global scale could be cross-verified by the mud reservoir beneath Utopia Planitia, which represents a significantly larger body of water at depths extending up to 7.2 km [39]. In addition, the estimated water volume may be overstated if the porosity is not fully saturated or if pre-existing water in the crust is not accounted for.

### Implications on the timing and pathways of water infiltration

While numerous studies have examined how water was stored in the Martian crust, discussions on the pathways through which water enters the crust remain limited. On Earth, subduction tectonics plays a key role in transporting large volumes of liquid water from the surface into the deep crust and the mantle [40]. In contrast, Mars lacks the complex system of interacting plates found on Earth. Instead, it has a stationary, thick and rigid outer layer, which has hindered the development of plate tectonics. Consequently, on Mars, it is not possible to hypothesize the transport of large volumes of liquid water (∼710–920 m GEL) from the surface to the base of the upper crust through subduction processes. Several pathways for water entry in the crust have been proposed, including crustal hydration and percolation of water, which ends up being trapped in pores and fractures. However, any hypothetical percolation from the surface through fractures and pores is precluded in modern Mars by the fact that, in the uppermost 5 km from the surface, water can only exist in the form of ice (Fig. 4a). This limitation did not exist during the Noachian when higher thermal gradients allowed the presence of liquid water throughout the entire upper crust (Fig. 4a).

In this context, we propose that liquid water penetrated from the surface to the base of the upper crust during the Noachian, through the dense network of ancient fractures created by intense Noachian bombardment, and before the subsequent decrease in temperature that prevented the presence of liquid water in the most superficial part of the Martian crust. Once created, these fractures may have been subsequently reactivated by planetary contraction due to Mars’ cooling. Mechanical studies of the Martian crust typically revealed a 10-km-thick layer of highly permeable fractures resulting from heavy impacts [19]. Modeling of lithospheric contraction suggests that fractures could extend downward to ∼10 km or deeper [41], but their existence at greater depths is, however, limited by lithostatic pressure [33]. The preservation of the fracture network may have been ensured over time by the secular cooling of the planet, whose contraction could have led to the formation of new fractures or the reactivation of old ones [41–43]. While pathways to create reservoirs of liquid water at the base of the upper crust may have been available for most of Mars history, the subsequent decrease in Martian temperatures would have prevented their use, due to the presence of water ice in the uppermost crust.

## CONCLUSION

We use the receiver functions from two impact events and one marsquake with high-frequency content to obtain the high-resolution shear wave velocity of the Martian upper crust. Three distinct layers are identified. The first layer, extending to a depth of 0.8 km, is likely composed of highly fractured material, or a mixture of ejecta, sediments and altered basalts. The second layer, a high-velocity zone reaching a depth of 5.4 km, suggests the presence of lithified sedimentary rocks or altered igneous rocks. The third layer with its lower boundary at a depth of 8 km exhibits a prominent low-velocity feature, interpreted as a high-porosity, water-saturated zone. The temperature and pressure conditions at this depth allow water to exist as liquid rather than ice. Considering the porosity of 20%–30% and assuming a global upper crust structure similar to that observed beneath the InSight lander, the liquid water volume preserved at the upper crust base ranges from 520 to 780 m GEL. This overlaps with the remaining liquid water volume of 710–920 m GEL, as estimated by other multidisciplinary studies. Our results provide the first seismic evidence of liquid water at the base of the Martian upper crust, shaping our understanding of Mars water cycle and the potential evolution of habitable environments on the planet. Such findings could be corroborated by future missions equipped with seismometers, designed to gather more detailed data at Elysium Planitia, where the current InSight observations were made, and also at other potential landing areas across Mars.

## METHODS

### True-amplitude receiver function method

The classical receiver function method typically requires selecting a Gaussian width factor, which controls the signal's bandwidth [44]. We implement a true-amplitude receiver function based on iterative deconvolution in the time domain by using an appropriately selected Gaussian factor. The selection of the Gaussian factor requires the shape of the Gaussian function to align as closely as possible with the attenuation of high-frequency components. Based on this criterion, we select a Gaussian factor of 12 to encompass all primary bands of signal energy (see Fig. 3), thereby enhancing the vertical resolution of crustal structures. Additional receiver-function data-processing details are provided in Data S2.

### Receiver function inversion

Two inversion schemes—deterministic and statistical Bayesian—are employed to cross-validate the inverted upper crustal structures. Deterministic and fixed-dimensional Bayesian inversion are two distinct approaches. Deterministic inversion focuses on identifying a single optimal model that minimizes the discrepancy between observed and predicted data. The scheme is implemented using a non-linear trust-region optimization algorithm [45]. In contrast, the fixed-dimensional Bayesian inversion approaches handle the inverse problem probabilistically, seeking to characterize the entire range of models consistent with the data [46]. As in Fig. 2a, the final receiver function for inversion is windowed from 0.25 s before to 3.0 s after the P-wave onset to exclude potential reverberations and later conversions from the middle crust and Moho. We invert the upper crustal structure down to a depth of 10 km. The initial model has a constant velocity of 1.7 km/s and the depth interval is set to 1 km. The thickness of each layer is variable during the inversion. The detailed model parameterization and inversion implementations are given in Data S3, and their validations via the synthetic example are in Data S4.

## Supplementary Material

nwaf166_Supplemental_File
