# Supplementary material for: Seismic evidence of liquid water at the base of Mars’ upper crust
Source: Natl Sci Rev. 2025 Apr 25;12(6):nwaf166. doi: 10.1093/nsr/nwaf166 (PMC12125978; doi:10.1093/nsr/nwaf166)
Supplement: nwaf166_Supplemental_File [file nwaf166_supplemental_file.docx]

**Supplementary data for**

**Seismic evidence of liquid water at the base of Mars' upper crust**

Weijia Sun (孙伟家)^1*^, Hrvoje Tkalčić^2^, Marco G. Malusà^3^, Yongxin Pan (潘永信)^1^

1 Key Laboratory of Planetary Science and Frontier Technology, Institute of Geology and Geophysics, Chinese Academy of Sciences; Beijing 100029, China.

2 Research School of Earth Sciences, The Australian National University; Canberra, ACT 2601, Australia.

3 Department of Earth and Environmental Sciences, University of Milano-Bicocca, 20126 Milan, Italy.

*Corresponding author.

WS: swj@mail.iggcas.ac.cn (email), 15801359580 (tel), 010-62010846 (fax)

**Supplementary Text**

**S1. Spectral analysis**

The Marsquake Service categorizes marsquakes into low-frequency (LF) and high-frequency (HF) families based on their frequency content, further classifying them into subcategories (Giardini et al., 2020). For instance, the LF family comprises two types: low-frequency (LF; < 1 Hz) and broadband (BB; predominantly < 2.4 Hz). Previous studies have primarily utilized LF marsquakes to investigate Mars' interior structure (Durán et al., 2022; Joshi et al., 2023; Knapmeyer-Endrun et al., 2021; Lognonné et al., 2020).

In this section, we compare the spectra of two impacts (S1000a, S0194b) and the largest tectonic marsquake (S1222a), all containing high-frequency components above 4 Hz, with a high-quality low-frequency (LF) marsquake (S0173a). Figure S1 illustrates their waveforms and scaleograms for the vertical component. The metadata of the events are listed in Table S1.

For the LF marsquake S0173a, the P-wave signal's spectral strength significantly exceeds the noise level at frequencies below 1 Hz. In contrast, for our selected three events (S1000a, S1094b, S1222a), the P-wave signal's spectral strength is substantially stronger than noise at frequencies up to 4 Hz and beyond. Notably, the spectrum of S1222a shows P-wave signal strength consistently exceeding noise levels across the seismometer's entire frequency range (0-10 Hz). The presence of these high-frequency components potentially enhances the vertical resolution of receiver functions.

**Table S1**. Metadata of marsquakes and impact events used in this study. LF: low frequency, BB: broadband

| Event name | Type | Quality | Baz (°) | Dist (°) | P (PP) onset |
| --- | --- | --- | --- | --- | --- |
| S0173a | LF | A | 90.98 | 30.6 | 2019-05-23 02:22:59 |
| S1000a | Impact | A | 34.0 | 126.1 | 2021-09-18 18:01:47 |
| S1094b | Impact | A | 51.4 | 58.5 | 2021-12-24 22:45:09 |
| S1222a | BB | A | 100.97 | 37.0 | 2022-05-04 23:27:45 |

**
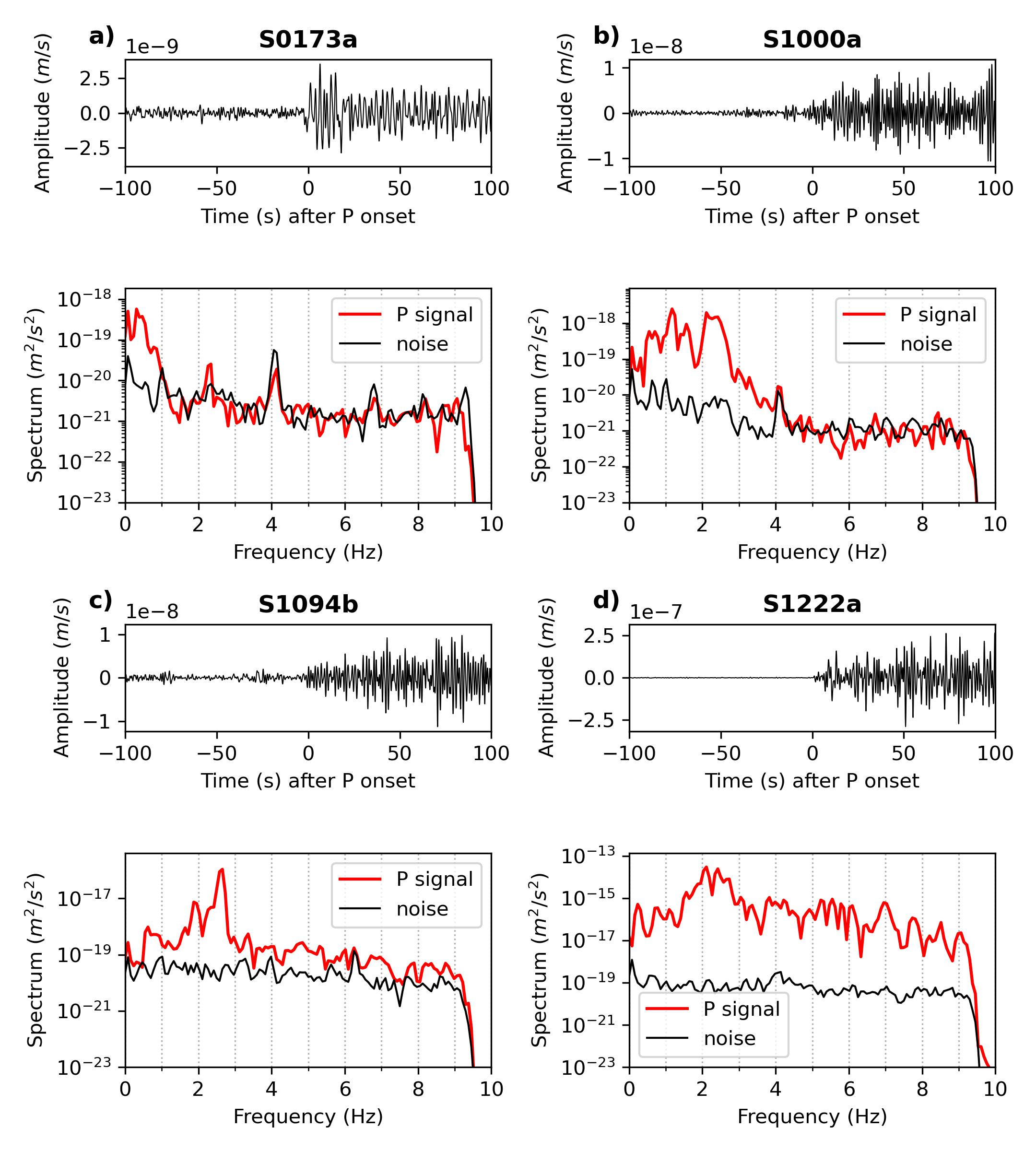
**

Figure S1. Waveform and spectral analysis for a) S0173a, b) S1000a, c) S1094b and d) S1222a. In each subfigure, the upper panel displays the vertical component waveform around the P-wave onset, while the lower panel presents the spectrum of the P-wave signal (red line) and noise (black line).

**S2. Data processing**

**S2.1 Basic processing**

The original data were recorded in three components: U, V, and W. Glitches were initially removed using a deep-learning tool (Xu et al., 2022). Subsequently, the instrumental response was removed to obtain velocity, and a pre-filter of (0.01, 0.05, 9, 10) Hz was applied. Finally, we rotated the UVW components to the conventional ZNE (vertical, north-south, east-west) components. Given the rich high-frequency content of the selected events, we employ the 00.HH(UVW) channels with a sampling rate of 100 Hz, rather than the 02.BH(UVW) channels used in previous studies.

**S2.2 Calculation of receiver function**

The receiver function (RF) was calculated using iterative deconvolution in the time domain, implemented through the *rf* package (Eulenfeld, 2020). The calculation of receiver functions (RFs) may vary due to the use of different time windows (Shi et al., 2023). We compute RFs using various time window lengths, maintaining a fixed starting time of 10 s before the P-wave onset. The mean RF is taken as the final RF. Figure S2 illustrates the mean RFs calculated using different time windows. The window ending range of 15–60 s indicates that RFs are computed in time windows ending between 15 s and 60 s after the P-wave onset, with a 1-s interval (Shi et al., 2023). This procedure generates 46 RFs. As seen, the averaged RF is stable, displaying similar phases across different time windows.

We calculate the mean RFs using different time windows for the three selected events. To ensure the best coherence among these events, we compute the normalized cross-correlation coefficient (CC) between them and sum the results, as shown in Figure S3, for each window ending range. We find that the window ending range of 15–120 s yields the highest CC, and is therefore selected for further analysis.


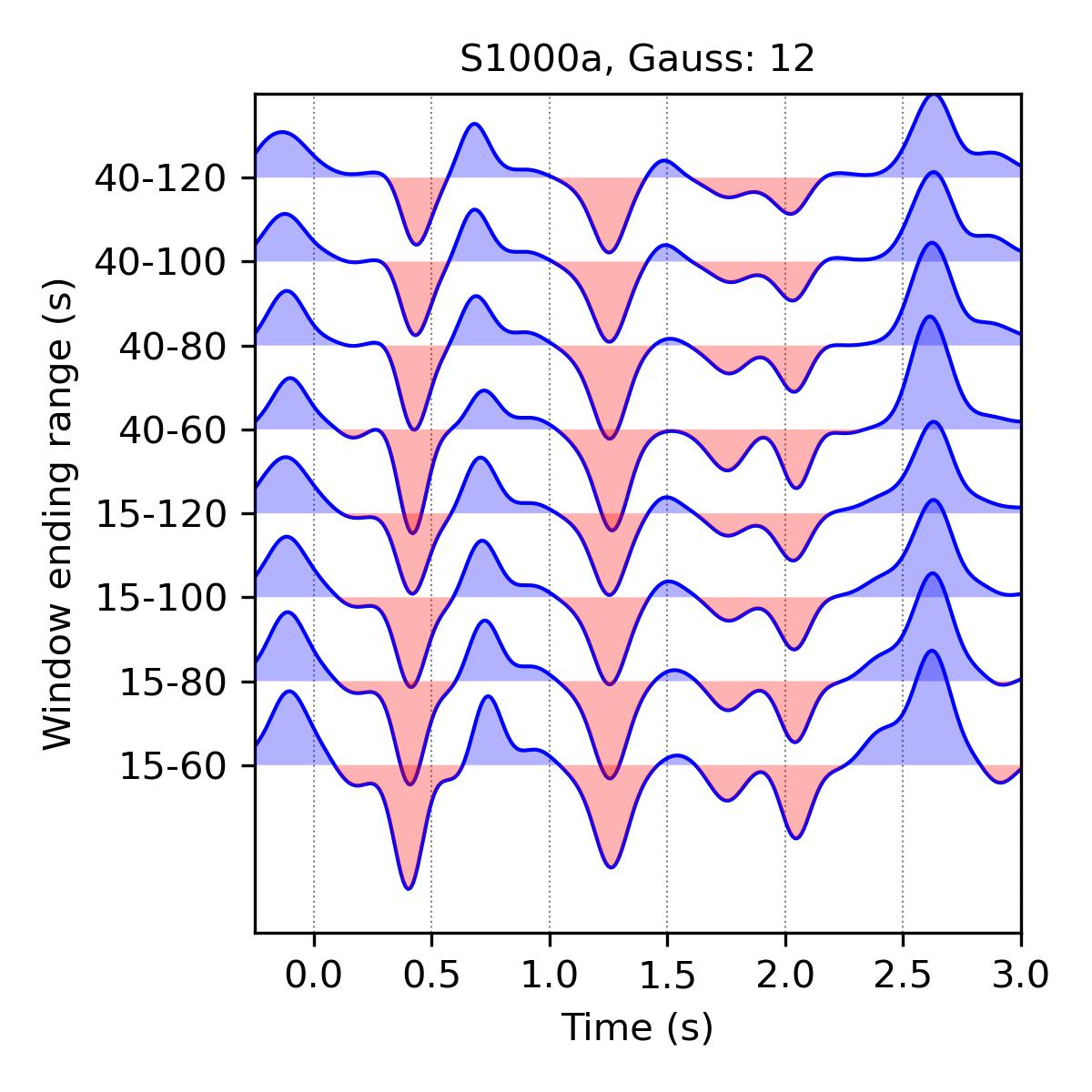


Figure S2. Tests of the time window length influence on receiver function calculation. Receiver functions for S1000a with a Gaussian factor of 12 are shown as an example.


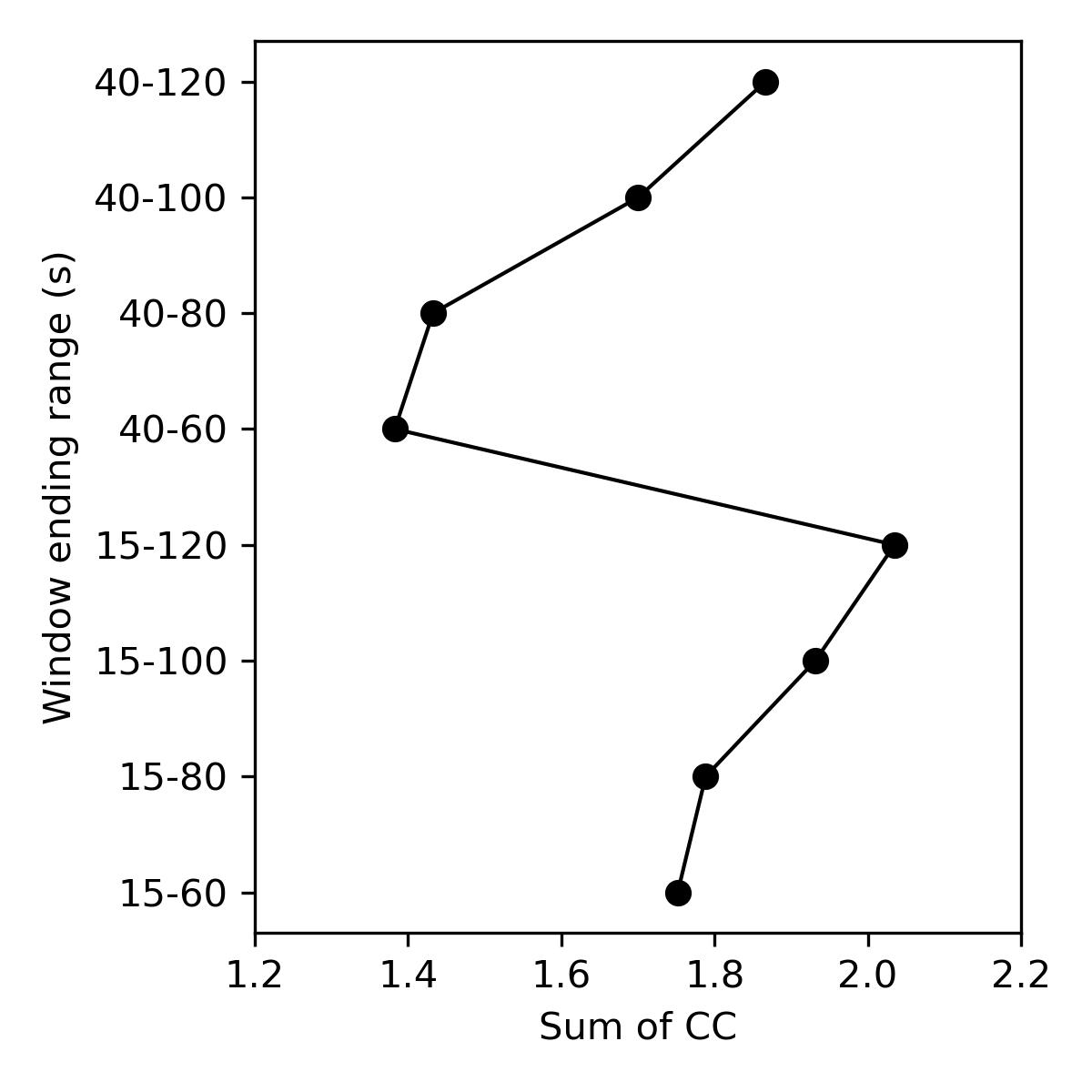


Figure S3. Selection of time window ending range.

**S2.3 Enhanced signals**

In this section, we compare the computed RFs using two Gaussian factors: 4 (Shi et al., 2023) and 12 (this study) as illustrated in Figure S4. As seen, while the phases are generally similar, the early phases (mostly <1.0 s) are significantly enhanced (indicated by black arrows) when using a Gaussian factor of 12, due to the inclusion of more high-frequency content.

**
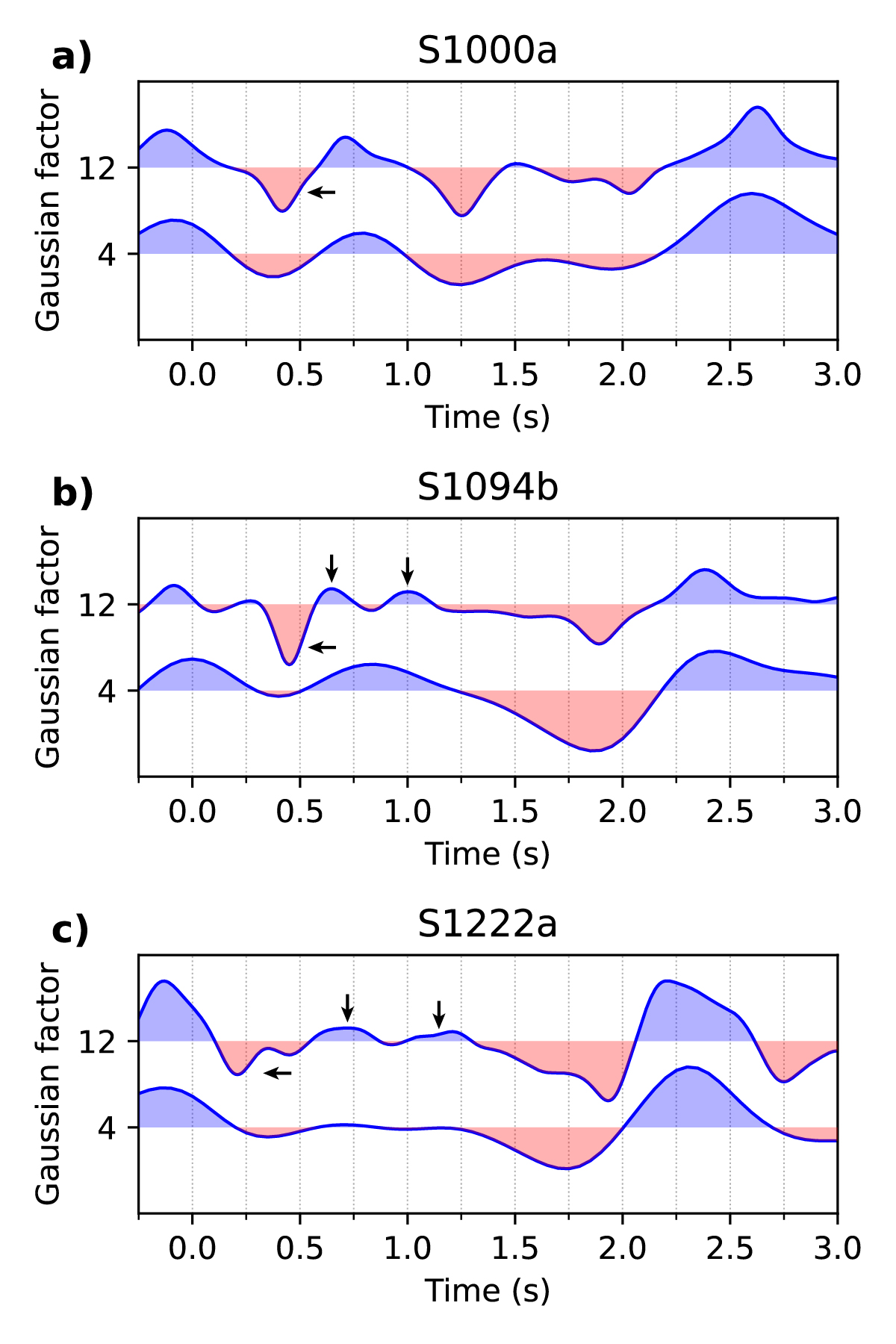
**

Figure S4. Comparison of receiver functions using Gaussian factor of 4 and 12 for a) S1000a, b) S1094b, and c) S1222a.

**S2.4 Stacked receiver function for inversion**

On Earth, receiver functions are typically calculated from earthquakes with magnitudes greater than 5.5, which thus provide a significantly high signal-to-noise ratio (SNR). Considering that marsquakes are generally of smaller magnitude, we would fit the stacked receiver function after moveout correction in this study. This scheme inverts the SNR-enhanced and stacked receiver function from three events.

Once the receiver functions for the selected events from the same station are obtained, we apply a moveout correction for a ray parameter of 0.10 s/km, which is an average derived from the selected events. This ray parameter is calculated using the KKS model obtained from the InSight data (Khan et al., 2021; Knapmeyer-Endrun et al., 2021; Stähler et al., 2021). After this correction, we stack the corrected receiver functions to obtain the final receiver function within the time window of -0.25 s to 3.0 s for inverting the upper crustal velocity structure. The workflow is shown in Figure S5.

**
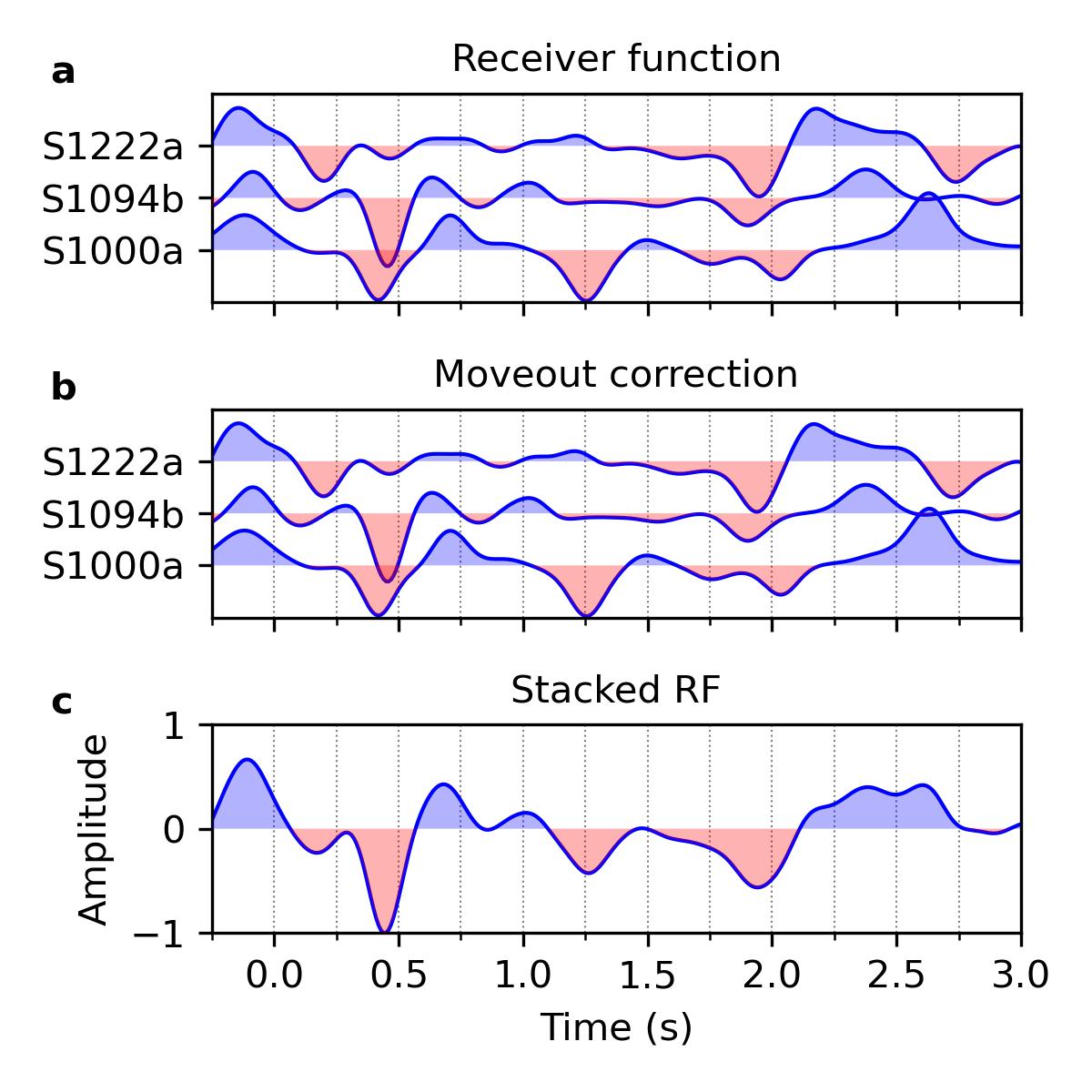
**

Figure S5. Workflow to obtain the finally stacked receiver function for inversion.

**S2.5 Length of the receiver function for inversion**

We carefully select a receiver function time window spanning from 0.25 s before to 3 s after the P-wave arrival. A longer time window risks including reverberations between the free surface and the upper crust's base for high-frequency contents. Since our objective is to image the velocity structure of the upper crust, our criterion is to include the Ps conversion from the upper crust's base, which inherently captures signals originating from its internal structures.

The Ps conversion from the base of the upper crust occurs at 2.4 s, as revealed by low-frequency (<1 Hz) receiver functions (Joshi et al., 2023). In our high-frequency receiver function analysis, this conversion occurs within the time range of 2.1–2.7 s. Based on these findings, we truncate the receiver functions to a time window spanning from -0.25 s to 3 s relative to the P-wave arrival for receiver function inversion.

**S3. Inversion of high-frequency receiver function**

**S3.1 Model parameterization**

In both inversion schemes— deterministic and the fixed-dimensional Bayesian—we fit the data using the same parameterization. We describe each layer with two parameters: layer thickness and shear-wave velocity. A previous study using low-frequency receiver functions revealed that the upper crust has a thickness of approximately 8 km and provided an averaged shear-wave velocity of 1.7 km/s (Joshi et al., 2023). Considering the existence of gradual discontinuities (more descriptions in S3.3), we employ an initial model with ten sub-layers, each having a thickness of 1 km, which can also help to reduce the inversion complexity. The ten-layered upper crust model is then connected to a half-space model. However, we do not fix the velocity and thickness of each sub-layer in the upper crust during both inversion schemes. For gradual interfaces, several thin layers can effectively mimic the gradual transition. For thick layers, multiple sublayers with similar velocities can represent a single thick layer. We set the velocity of all sub-layers to be 1.7 km/s. This model setting balances both model smoothness and vertical velocity variations.

**S3.2 Regularization in the two inversion schemes**

The Bayesian inversion aims to estimate the probability distribution of the parameters given the data and a prior. The regularization concept in this scheme is naturally handled by the prior distribution. We here mainly focus on the regularization used in the classical deterministic approach for the actual data. Further investigations are performed in below Section 4 via synthetic data.

In the deterministic inversion, the regularization, i.e., damping factor, is applied to prevent excessive changes in the velocity model during the analysis. In this study, the influence of the damping factor is examined by selecting various damping factors, including 0 (representing no damping), 1, 10, 100, 1000, and 10000, each corresponding to different levels of damping strength, from none to weak and strong. Figure S6 illustrates the inverted models derived from various damping factors. For smaller damping factors, the inverted models exhibit variability and consistency across each sub-layer. However, with larger damping factors—particularly at the highest factor of 10,000—the inverted models show significantly less variation or remain nearly constant throughout the depth.

The application of regularization aids in stabilizing the inversion process; however, it can also notably diminish the resolution of the inverted model (Sun & Kennett, 2016). This observation is supported by our test, as shown in Figure S6. Keeping in mind that our goal is to enhance vertical resolution, we opted for smaller damping factors, specifically 1 and 10. Favorably, the inverted models resulting from these smaller damping factors were corroborated by the models derived from a distinct Bayesian scheme. In addition, the models inverted from smaller damping factors consistently present the low-velocity zone at the base of the upper crust, well matching the model obtained from the Bayesian inversion.


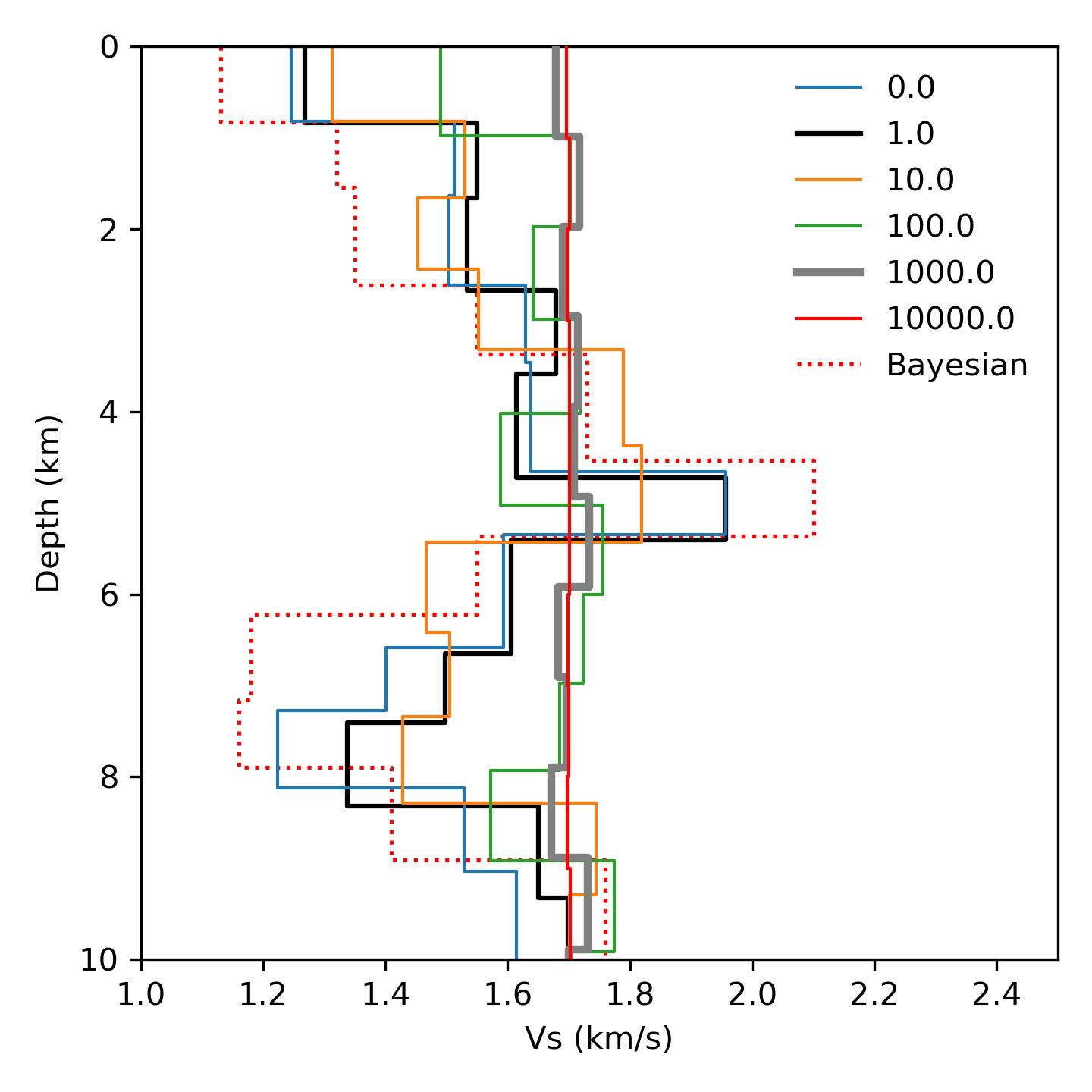


Figure S6. Inverted S-wave velocity models from the deterministic scheme with various damping factors. The factor of zero means no damping is applied. The mean model of the Bayesian inversion is solely shown for clarity. The probability density function (PDF) of the Bayesian models is illustrated in Figure 2a.

**S3.3 Bayesian inversion**

Rather than focusing solely on the optimal solution, Bayesian inversion seeks to characterize the uncertainty in the parameter estimates through the use of probability distributions. Trans-dimensional Bayesian inversion is a good choice for unknown structures. However, the algorithm may over-fit the data and add more layers than necessary when the noise is underestimated (Bodin et al., 2012). As the Martian noise feature is not well known, to avoid the potential data overfitting, we adopt a fixed-dimensional inversion approach using the same model parameterization (see S3.1) as in the deterministic inversion for consistency. Since both velocity and thickness are allowed to vary during the inversion, gradual discontinuities can be effectively represented by a few sub-layers (piecewise constant models) over a broad depth range, while sharp discontinuities are captured by significant velocity increases within a narrow depth range.


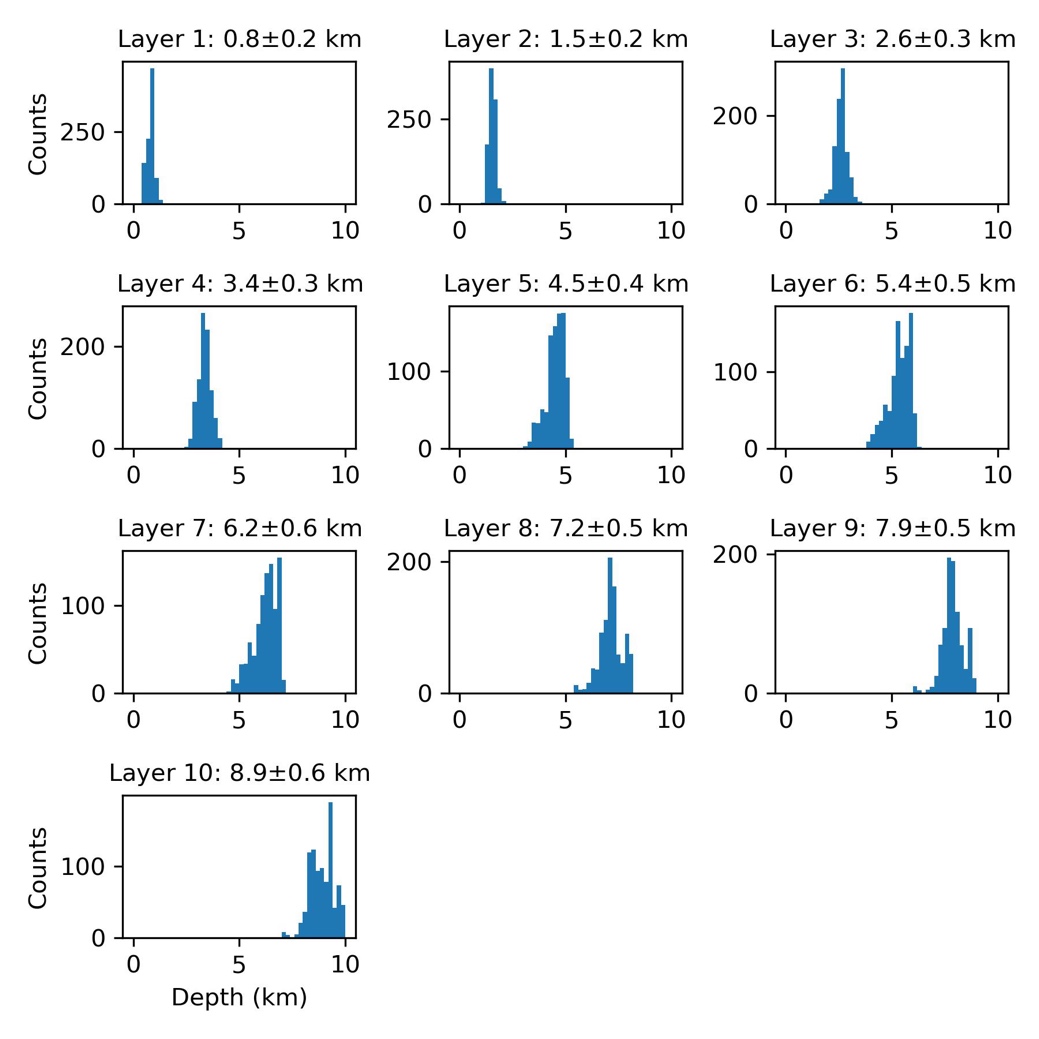


Figure S7. Depth distributions of each sub-layer in the upper crust. The layer number, along with the corresponding mean depth and standard deviation, are provided. The posterior distribution is far from a unimodal Gaussian distribution because the receiver function (RF) inversion is highly nonlinear (Bodin et al., 2012).

Figure S7 illustrates the depth distributions of each sub-layer within the upper crust. The standard deviation increases from 0.2-0.3 km to 0.5-0.6 km as the depth increases. This change in standard deviation with depth may indicate that the discontinuities become a little more gradual at greater depths. We, therefore, employ fixed-dimensional Bayesian inversion with a greater number of sub-layers to account for the possibility of gradual velocity variations.

**S3.4 Residual waveform**

To determine the detailed shear-wave velocity structure of Mars' upper crust, we conducted an inversion analysis on the high-frequency receiver functions derived from three seismic events. Given that the quantity of receiver functions utilized in this study is considerably less than those typically employed in terrestrial investigations, we have undertaken additional validation measures. This involves comparing the residual waveforms—defined as the difference between predicted and observed waveforms—for both the deterministic and Bayesian inversion models. This examination ensures the reliability of our findings despite the limited dataset.

Figure 8a depicts the observed receiver function (blue line) alongside the predicted receiver function from the deterministic-inverted model (thin black line). These are superimposed over the probability density function of the receiver function ensemble derived from the Bayesian models. Also shown are the mean receiver function (thick black line) and the 1-standard deviation range (black dashed lines).

The corresponding residual waveforms are illustrated in Figure 8b. As seen, the residual waveforms from various models predominantly fall within the 1-standard deviation (dotted lines) range of the receiver functions predicted by the Bayesian models. Notably, the residuals for the mean model are minimal, with the exception of some clear bias around 0 seconds. This bias may be attributed to the presence of highly variable, thin, multiple sediment layers (Hobiger et al., 2021; Mun & Kim, 2024), which can be as thin as 100 meters or less, beyond the resolution capabilities of our data.


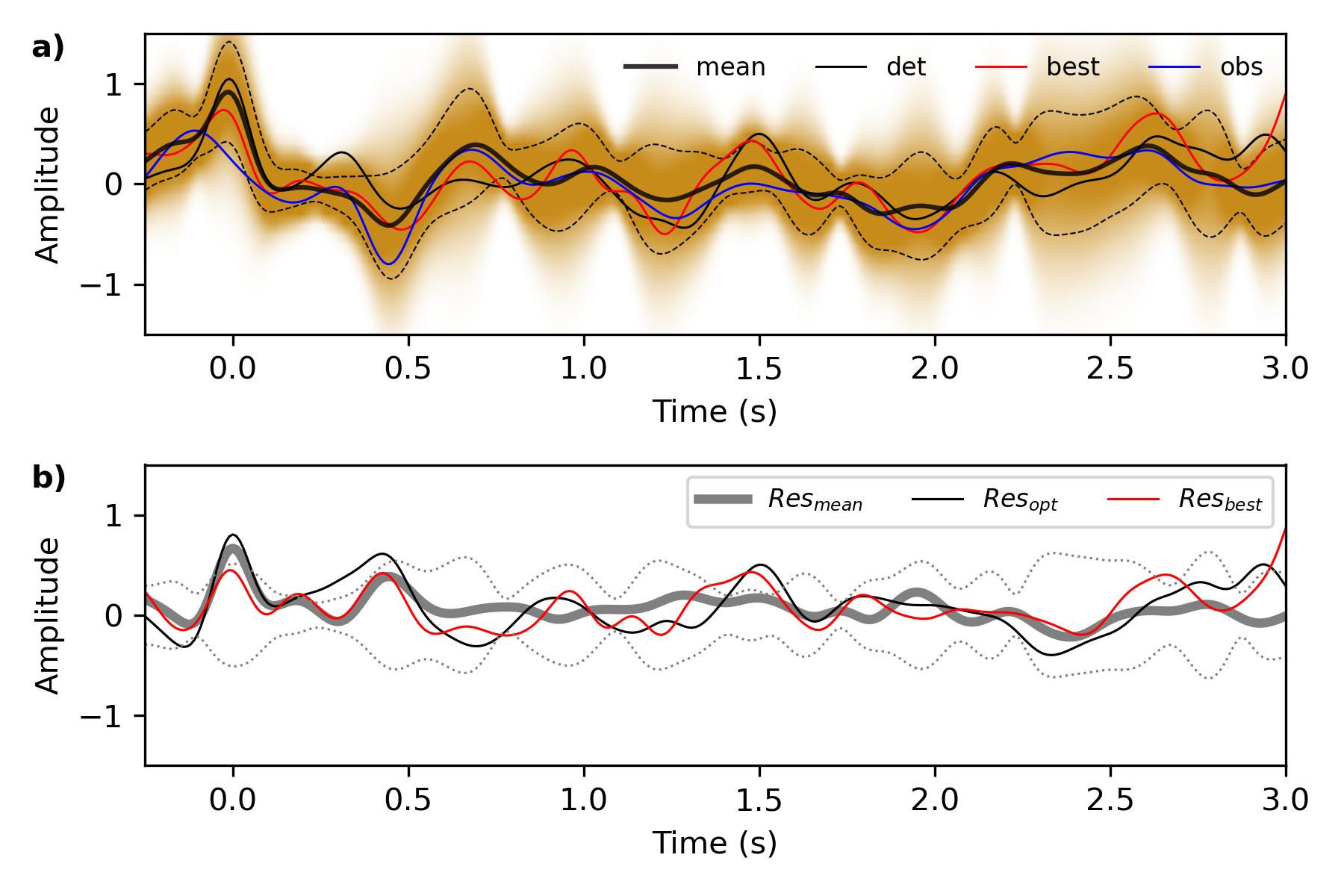


Figure S8. a) Receiver functions calculated from the inverted models using both deterministic (det) and Bayesian (mean, best) methods. b) The differences between the predicted receiver functions and the observed (obs) receiver functions. Additional details are provided in the text.

**S4.** **Validation of inversion via synthetic data**

In this section, we further validate the implementation of the two inversion schemes by designing a three-layered model based on the inversion results. The first layer has a thickness of 0.8 km and a low velocity of 1.1 km/s. The second layer, with a thickness of 4.6 km, exhibits a high velocity of 1.7 km/s. The third layer, representing the base of the upper crust, has a thickness of 4.0 km and a velocity of 1.3 km/s. This designed model is referred to as the "true model" because its structure is well-defined and known. The true model is shown in both Figures S9a and S9c. We synthesize the corresponding receiver function for the true model to serve as the observations. We then apply both the deterministic and fixed-dimensional Bayesian inversion schemes to evaluate their performance in recovering the true model using the same parameterization as the actual data.

**S4.1 The deterministic inversion**

In Figure 9, we compare the inverted models from the two schemes (colorized thin lines) with true models (black thick lines). Figure 9a presents the models with varied damping factors from the deterministic scheme with various damping factors. The model with the largest damping factor of 1000 does not clearly show velocity anomaly. As the damping factors decrease, the inverted models converge to the true model, similar to the inversion of actual data tested in Section 3.2.

Figure 9b displays the calculated receiver functions derived from the models shown in Figure 9a. The calculated receiver functions for various damping factors, except for the largest damping factor of 1000 (blue line), correlate well with that of the true model (black line). This indicates that the inverted models from the deterministic scheme can effectively recover the pattern of velocity anomalies. However, these models exhibit slight deviations from the true model, as illustrated in Figure 9a. This may reflect the non-uniqueness commonly associated with deterministic inversion methods.

**S4.2 The Bayesian inversion**

Figures 9c and 9d illustrate the inverted models from the fixed-dimensional Bayesian inversion and the corresponding calculated receiver functions, which are compared to those of the true models. In Figure 9c, the mean model (red line) and the probability density function (PDF, orange shading) of the inverted models from the Bayesian inversion closely align with the true model (black line). This near-perfect match indicates a high degree of accuracy in the Bayesian inversion method. Additionally, the correlation between the Bayesian model and the true model is notably stronger than that observed in the optimized models, highlighting the superior performance of the Bayesian approach in recovering the true subsurface structure, at least for this test in this section.

**S4.3 Validation of determining the number of sub-layers in the upper crust**

The receiver function inversion is highly nonlinear (Bodin et al., 2012). To reduce the complexity of the inversion process, we adopt a fixed number of layers for both inversion schemes. To balance this complexity with the accurate representation of discontinuity sharpness (whether sharp or gradual), we use 10 layers with varying thicknesses and velocities in each sublayer. For gradual interfaces, several thin layers can effectively mimic the gradual transition. For thick layers, multiple sublayers with similar velocities can represent a single thick layer.

We verify the model setting using synthetic data, as illustrated in Figure S9, with the same model parameterization as the actual data. As seen, the inverted models in Figures S9a and S9c accurately describe the true velocity model. The first layer, the thinnest, of the upper crust is represented by a single sub-layer. The second layer, the thickest, is described by five sub-layers with similar velocities, suggesting a nearly constant velocity profile. The third layer, of moderate thickness, is represented by four sub-layers. Similar to the second layer, these sub-layers exhibit minimal velocity perturbations. With this test based on synthetic data, our two carefully designed inversion schemes are proven to be valid and effective.

**S4.4 Vertical resolution of receiver function inversion**

Vertical resolution is intrinsically linked to the frequency content used in the analysis. In our case, with a frequency of 4 Hz and a velocity of 1.7 km/s, the wavelength—and thus the vertical resolution—is 0.425 km. However, the high-frequency component up to 4 Hz is weak, as demonstrated in Figure S10. The dominant frequency is approximately 2.5 Hz (Figure S10), resulting in a resolution of 0.68 km. So the potential resolution ranges 0.425–0.68 km. This represents the theoretical calculation of vertical resolution.

In Figure 2, the inverted models have ten layers, which are inherited from the initial model setting as described in Section S3.1. Given that we use only three events—compared to tens of events typically analyzed on Earth—we refrain from overinterpreting these ten layers. Conservatively, we interpret the layering within the upper crust based on significant velocity variations observed at depths of 0.8 km, 5.4 km, and 8 km, which is verified in Section S4.3.

To evaluate resolution, we conducted a numerical test by designing a layer with varying thickness. The first interface was set at a depth of 2 km, with a second interface beneath it at varying depths. If the converted phase (P_2_s) from the second interface can be distinguished from the phase (P_1_s) of the first interface, the receiver function can resolve the layer thickness for a given Gaussian factor.

For comparison, we selected two Gaussian factors: 6 and 12. As shown in Figure S11, for the lower Gaussian factor of 6, the lower limit of vertical resolution is 1 km. With the higher Gaussian factor of 12—corresponding to higher frequency content—the lower limit of vertical resolution improves to 0.5 km. Based on the signals in Figure 2a, we consider the structures derived from receiver function signals that originate from realistic geological scales larger than the theoretical resolution.


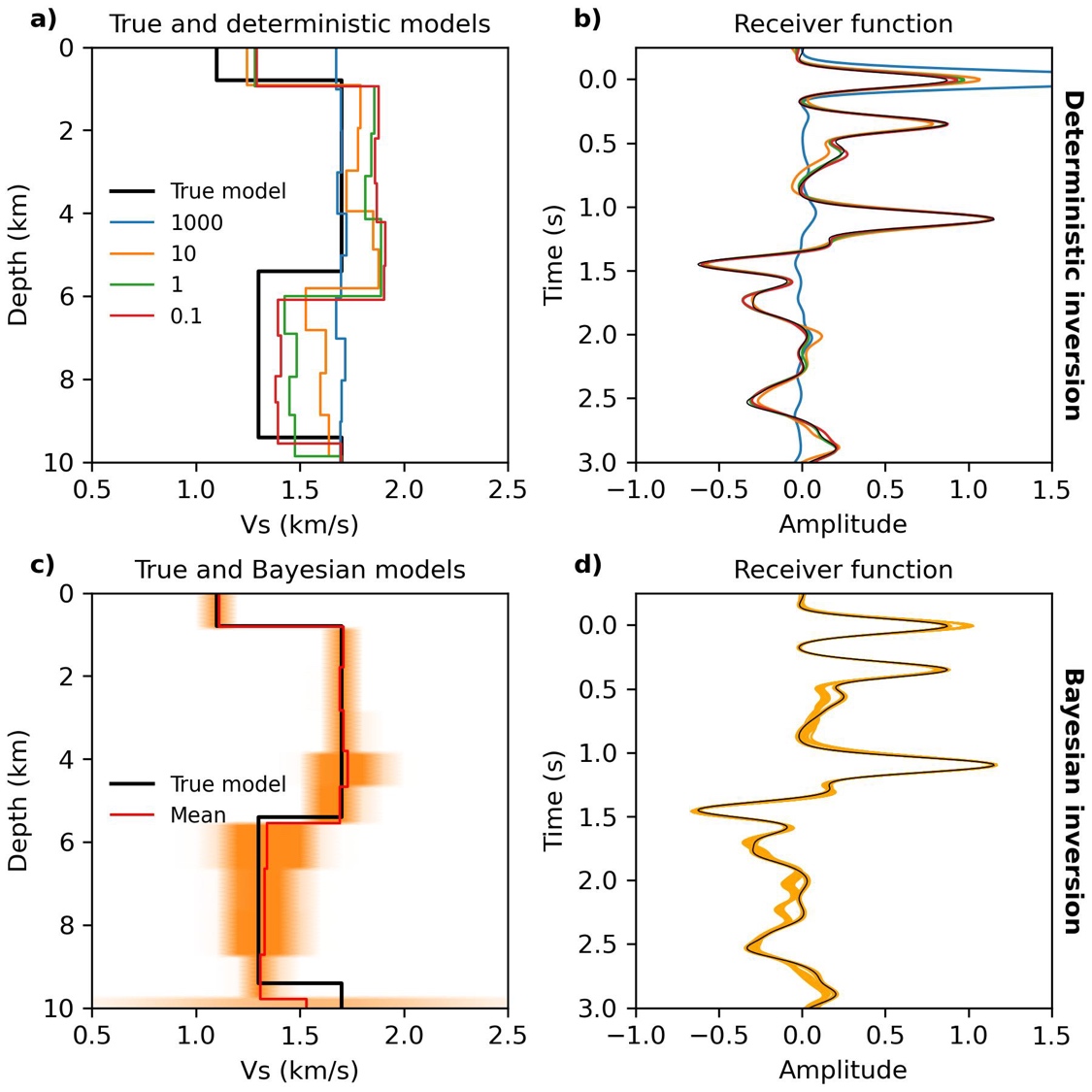


Figure S9. Validation of the deterministic and fixed-dimensional inversion via synthetic data. (a) Inverted models from the deterministic scheme and (b) the corresponding receiver functions. (c) and (d) same as (a) and (b), but for the Bayesian inversion.


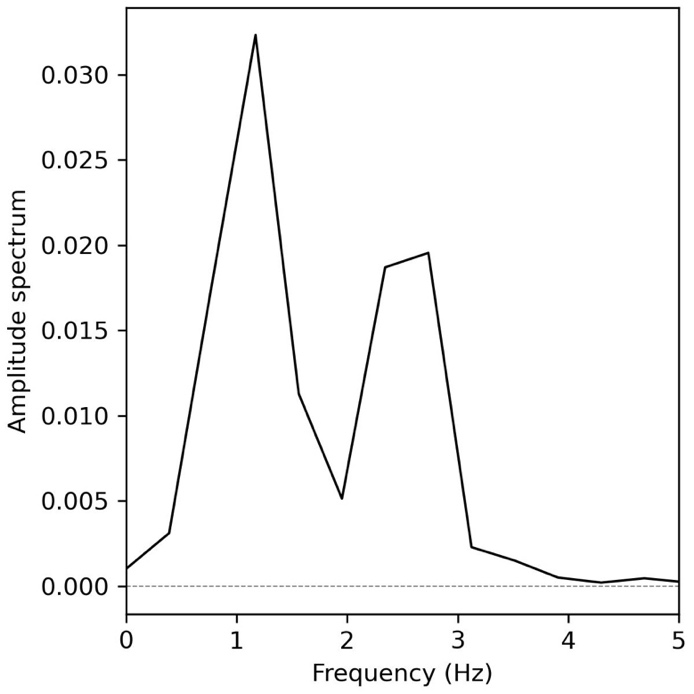


Figure S10. Spectrum of the stacked receiver function corresponding to the data presented in Figure 2a.


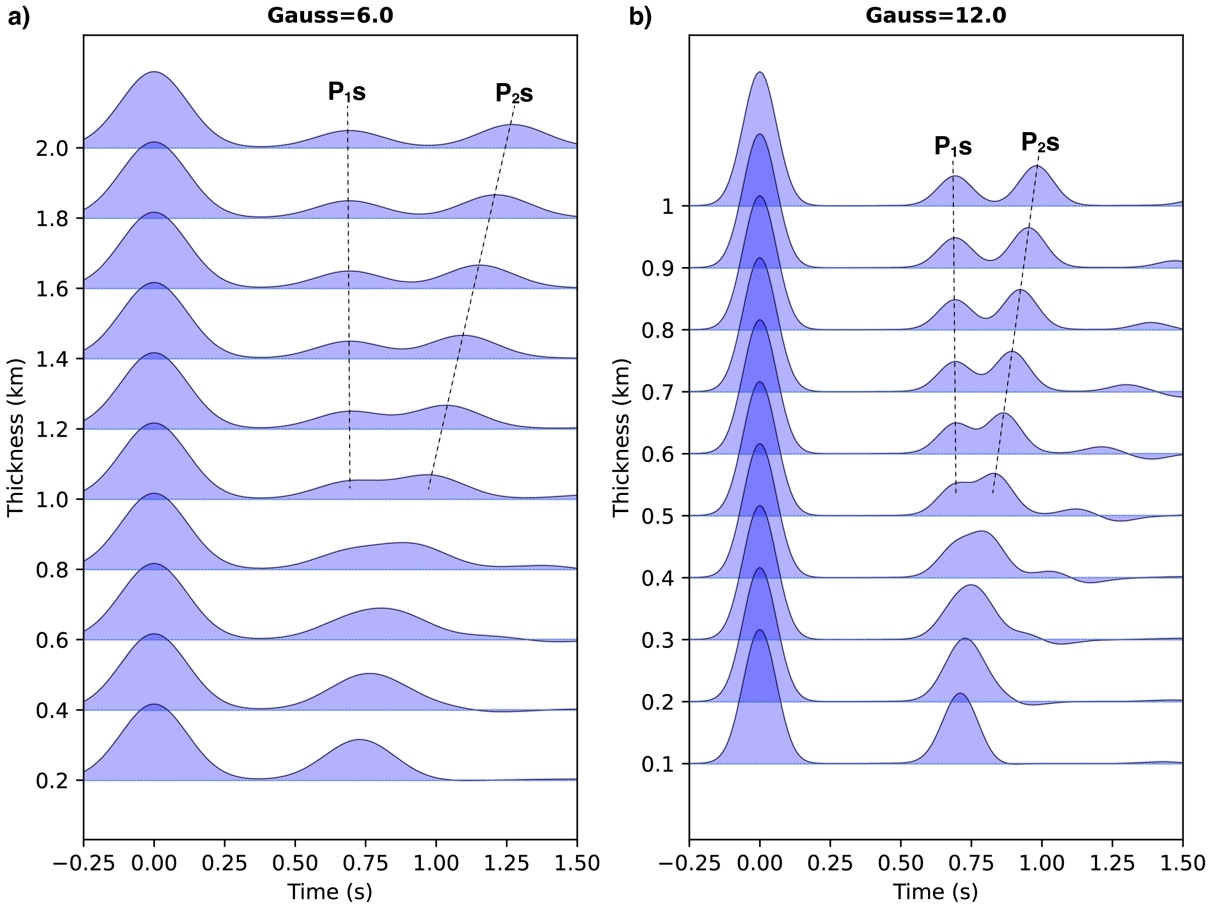


Figure S11. Examination of the vertical resolution of receiver function. a) Gaussian factor of 6 and b) Gaussian factor of 12.

**S4.5 Velocity inversion of individual receiver functions**

We select three events for receiver function inversion. There are two possible methods to invert velocity models: by fitting stacked receiver functions or by fitting individual receiver functions. The approach of stacking all receiver functions is more commonly employed for velocity model inversion. On Earth, it is standard practice to use tens of events with magnitudes greater than 5.5 to compute the stacked receiver function for a given station.

However, there is only one station on Mars, and the magnitude of the largest marsquake (S1222a) is 4.7. Although the signals from these marsquakes appear robust, their spectra exhibit significant variability, as shown in Figures 1b–1e. To ensure the reliability of the inverted velocity models, we further adopt the second strategy of inverting individual receiver functions for S1000a, S1094b, and S1222a. In Figure S12, the velocity models derived from individual receiver functions of these three events are compared with the model derived from the stacked receiver function. The blue-shaded region represents the low-velocity zone (LVZ), interpreted as containing liquid water. The LVZ is consistent across the model derived from the stacked receiver function (Figure S12a) and the models derived from individual receiver functions (Figures S12b–S12d). This consistency further demonstrates that the LVZ feature is stable and reliable.


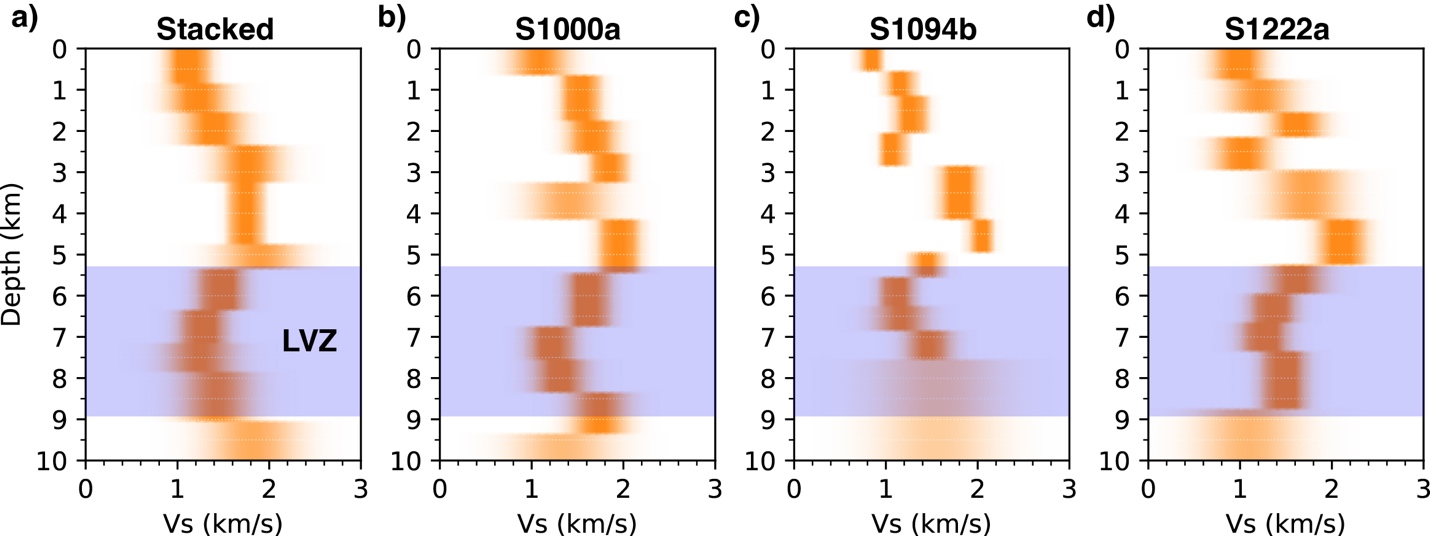


Figure S12. Inverted velocity models derived from (a) stacked receiver functions and individual receiver functions of three events: (b) S1000a, (c) S1094b, and (d) S1222a. The blue-shaded regions represent the low-velocity zone (LVZ).

**References**

Bodin, T., Sambridge, M., Tkalčić, H., et al. (2012). Transdimensional inversion of receiver functions and surface wave dispersion. *Journal of Geophysical Research: Solid Earth, 117*(B2).

Durán, C., Khan, A., Ceylan, S., et al. (2022). Seismology on Mars: An analysis of direct, reflected, and converted seismic body waves with implications for interior structure. *Physics of the Earth and Planetary Interiors, 325*.

Eulenfeld, T. (2020). rf: Receiver function calculation in seismology. *Journal of Open Source Software, 5*(48), 1808.

Giardini, D., Lognonné, P., Banerdt, W. B., et al. (2020). The seismicity of Mars. *Nature Geoscience, 13*(3), 205-212.

Hobiger, M., Hallo, M., Schmelzbach, C., et al. (2021). The shallow structure of Mars at the InSight landing site from inversion of ambient vibrations. *Nature Communications, 12*(1), 6756. <https://doi.org/10.1038/s41467-021-26957-7>

Joshi, R., Knapmeyer‐Endrun, B., Mosegaard, K., et al. (2023). Joint Inversion of Receiver Functions and Apparent Incidence Angles to Determine the Crustal Structure of Mars. *Geophysical Research Letters, 50*(3).

Khan, A., Ceylan, S., van Driel, M., et al. (2021). Upper mantle structure of Mars from InSight seismic data. *Science, 373*(6553), 434-438.

Knapmeyer-Endrun, B., Panning, M. P., Bissig, F., et al. (2021). Thickness and structure of the martian crust from InSight seismic data. *Science, 373*(6553), 438-443.

Lognonné, P., Banerdt, W. B., Pike, W. T., et al. (2020). Constraints on the shallow elastic and anelastic structure of Mars from InSight seismic data. *Nature Geoscience, 13*(3), 213-220. <https://doi.org/10.1038/s41561-020-0536-y>

Mun, E., & Kim, B. (2024). Constraining wave velocities for shallow depths on Mars. *Bulletin of the seismological Society of America, 114*(2), 673-689.

Shi, J., Plasman, M., Knapmeyer‐Endrun, B., et al. (2023). High‐Frequency Receiver Functions With Event S1222a Reveal a Discontinuity in the Martian Shallow Crust. *Geophysical Research Letters, 50*(5).

Stähler, S. C., Khan, A., Banerdt, W. B., et al. (2021). Seismic detection of the martian core. *Science, 373*(6553), 443-448.

Sun, W., & Kennett, B. (2016). Uppermost mantle P wavespeed structure beneath eastern China and its surroundings. *Tectonophysics, 683*, 12-26.

Xu, W., Zhu, Q., & Zhao, L. (2022). GlitchNet: a glitch detection and removal system for SEIS records based on deep learning. *Seismological Society of America, 93*(5), 2804-2817.
